# Material efficiency at the component level: how much metal can we do without?

**DOI:** 10.1098/rsta.2023.0245

**Published:** 2024-11-04

**Authors:** Julian M. Allwood, Omer Music

**Affiliations:** ^1^ Department of Engineering, University of Cambridge, Trumpington Street, Cambridge CB2 1PZ, UK; ^2^ Mechanical Engineering Department, TED University, Ankara, Turkey

**Keywords:** material efficiency, steel, aluminium, climate mitigation

## Abstract

Global production of steel and aluminium is a major driver of greenhouse gas emissions. Various processes might allow continued primary production of the two metals, but all depend on emissions-free electricity or carbon storage, and global capacity of these two key resources will be below demand for decades to come. As a result, zero-emissions steel and aluminium will mainly come from recycling, but supply will be lower than demand. This motivates demand reduction, and for the first time, this article estimates the inefficiency in current metal use by component type. The results demonstrate that around 80% of steel and 90% of aluminium liquid metal produced today may be unnecessary. Around 40% of liquid steel and 60% of liquid aluminium are never used in final components as they are removed along the supply chain of manufacturing. Of the metal that enters final service, approximately one-third could be saved by avoiding component over-specification. A further third could be saved, where the properties of metal are not used to their limits. These results point to specific opportunities for innovation in design and manufacturing technology, of which the highest priority is to re-think the use of sheet metal in construction.

This article is part of the discussion meeting issue ‘Sustainable metals: science and systems’.

## 1. Introduction: anticipating a metal supply shortage

The UK and many other countries have made legally binding commitments to have zero emissions by 2050. Most political effort to date, supported by the innovation and investment communities, has focused on supply-side interventions, in the hope that novel technologies can replace today’s emitting processes without substantial change to end-user experiences or the structure of the economy. However, these technologies are deploying slowly [1] as has always been the case [2–4], yet there is a wide and largely overlooked range of opportunities for innovation on the demand side.

**Figure 1.**
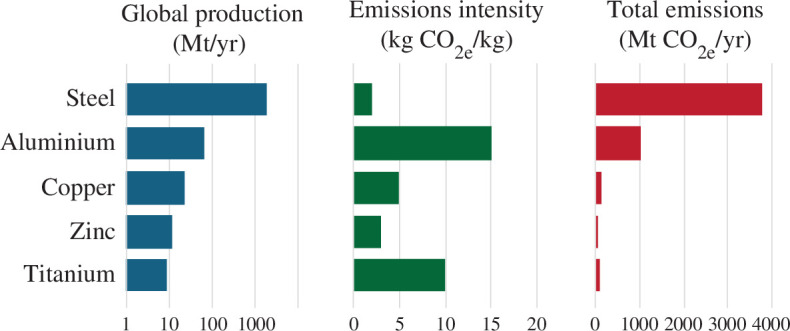
Production, emissions intensity (averaged across primary and secondary production) and global emissions of the five most-used metals (data sources: metals production volumes [7], emissions intensity [8–10]).

Around 78% of today’s global greenhouse gas emissions are caused by energy and industrial processes [5], a third of which are caused by industry; 56% of industrial emissions are caused by the production of just five materials, including steel and aluminium [6]. Figure 1 shows that of the five most important metals, copper, zinc and titanium have greater average emissions intensity than steel, but we use steel and aluminium in such volumes that these two metals dominate emissions from metal production. For almost all products, emissions from producing the liquid forms of these two metals exceed the emissions of all downstream processing.

Several options for supplying steel and aluminium with zero emissions have been developed and evaluated over recent decades. For steel, the main technologies are carbon capture, utilization and storage, hydrogen reduction, direct electrification and the use of bio-energy [11]. For aluminium, the options are decarbonization of the electricity supply, improved energy efficiency and the use of inert anodes [12]. In theory, many of them could operate at global scale. However, in practice, all of them depend on three fundamental resources: emissions-free electricity, biomass and carbon storage. For example, it is possible to reduce iron with hydrogen, but hydrogen must be manufactured, and in turn, if it is made without emissions, it depends on either emissions-free electricity or carbon capture and storage. The reality of these limits is reflected in some recent analyses on resource constraints for decarbonizing the foundation industries [13–15].

**Figure 2.**
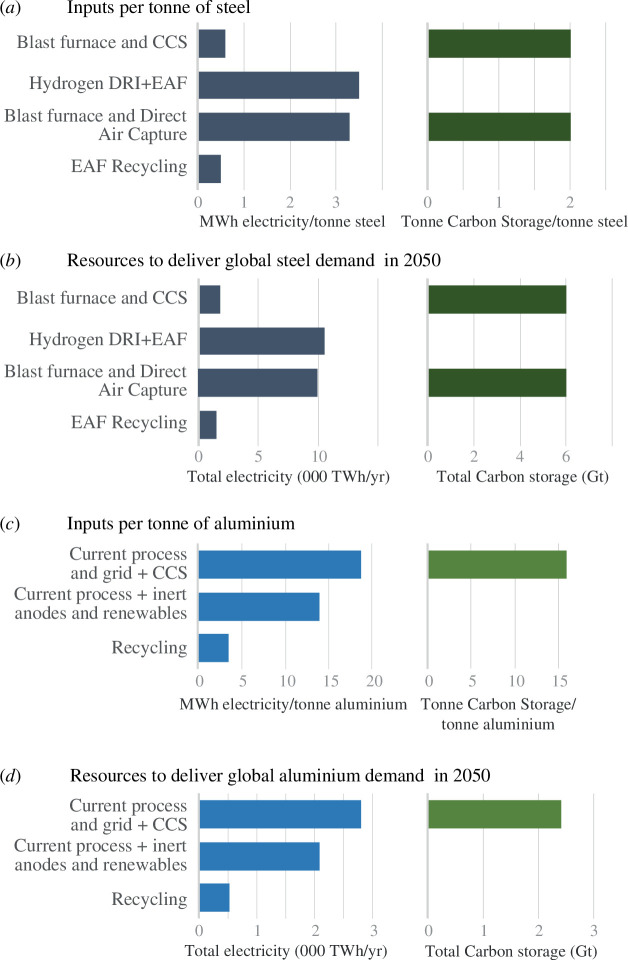
The options for producing (*a,b*) steel and (*c,d*) aluminium with zero emissions. (*a,c*) showing input requirements per tonne of metal and (*b,d*) anticipated demand for these inputs in 2050 if demand grows without climate constraints (Data sources: steel [11,16,17] aluminium [12] electricity requirement of CCS [18]).

Accordingly, the viable options for future emissions-free supply of the two metals are summarized in figure 2, which demonstrates the intensity of their demand for the two fundamental resources of emissions-free electricity and carbon storage.

Figure 2 begs the question, how much electricity and how much carbon storage will we have in 2050? Since the 1960s, global capacity for emissions-free electricity generation has grown approximately linearly, with a slight increase in gradient around 2010 as offshore wind generation expanded [19]. The current rate of growth is around 400 TWh yr^−1^. Projecting forwards, and anticipating that at most this rate might double, global emissions free generation in 2050 is likely to be in the range 20 000–30 000 TWh yr^−1^. Global carbon storage capacity has similarly grown linearly since 2010, at a consistent rate of 2Mt CO_2_ yr^−1^, to reach around 40 Mt CO_2_ yr^−1^ today, or around 0.08% of global anthropogenic greenhouse gas emissions (of which around 72% are carbon dioxide, which is the only greenhouse gas amenable to CCS processes). Again, assuming this rate might continue or at best double, global capacity in 2050 could be of the order of 100–160 Mt CO_2_ yr^−1^.

Assuming that the expanded supply of these two resources is shared among sectors in proportion to their current greenhouse gas emissions, we can forecast that by 2050, the global steel sector will be able to access 1700–2600 TWh yr^−1^ of emissions-free electricity with the aluminium industry having around one-tenth of that. It is very unlikely that either industry will be able to draw on any significant supply of carbon storage, as the global total will remain small, even if deployment rates increase by a factor of 10.

**Figure 3.**
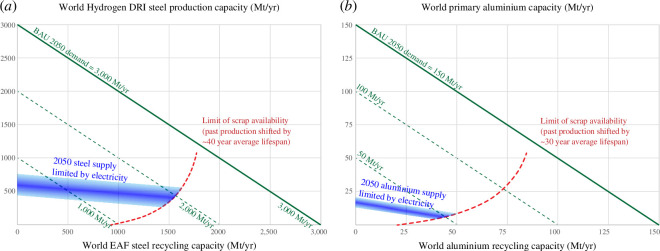
Projection of global supply and demand for (*a*) steel and (*b*) aluminium in 2050, with a realistic forecast of zero-emissions resource availability.

Reviewing the metal production options in figure 2, these realistic projections demonstrate that no primary metal process depending on carbon capture and storage will operate at significant scale by 2050. Instead, metals must be produced with electricity only. Applying the budget of emissions-free electricity to the processes of figure 2 leads to the prediction of figure 3: compared to the demand for the two metals that we would expect if economic development continued untroubled by climate change (taken from analyses of stocks and flows for steel [5] and aluminium [20]); by 2050, we will be able to supply only a small fraction of this demand without emissions.

The figure shows that there will be a substantial gap between emissions-free supply and demand for the two metals. If today’s political prevarication about mitigation continues, perhaps this supply will be augmented by continued supply from existing emitting assets. However, by 2050, it is likely that the impacts of climate change will be so severe that there will be a significant public pressure to adopt urgent and radical action. This article therefore continues on the premise that delivering zero emissions is a higher priority than the continued prosperity of today’s high-emitting industries.

There is high and unquantifiable uncertainty behind the forecasts in figure 3, but however this uncertainty plays out, the figure shows that a supply shortfall is virtually certain. Despite a current political culture of techno-optimism, it will in reality be difficult to expand the supply of either metal significantly so we must expect to live with much less metal than we have had before. This article therefore aims to take a new look at the technical potential for living well with much less metal.

## 2. Prioritization: how and for what is metal used?

**Figure 4.**
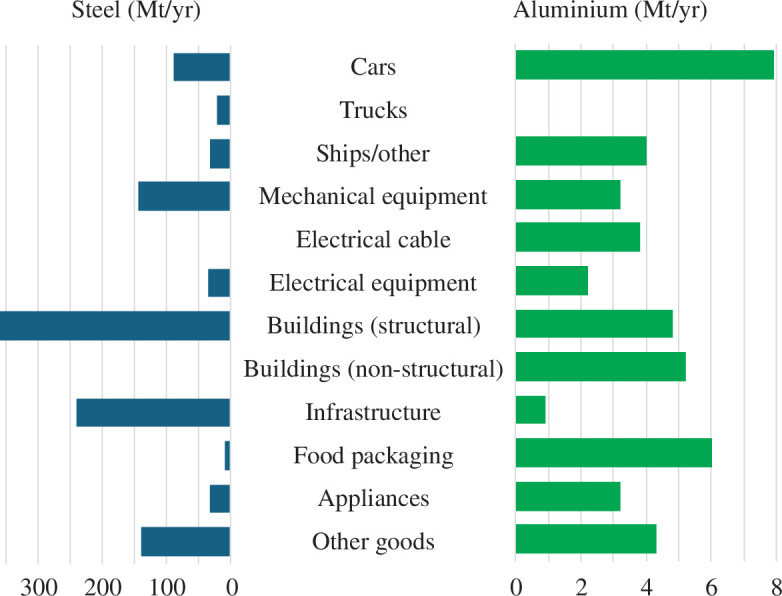
The use of steel and aluminium in products [21,22].

Because of the long supply chains required to convert new liquid metal into final products, there is no convenient source of data characterizing the final uses of steel and aluminium. Global Sankey diagrams revealing the ‘flow’ of the metals from ore or scrap through to final goods in 2008 have been constructed for steel [21]and aluminium [22], with some similar analyses providing national-scale data [23–25]. The global 2008 analysis [21,22] is used to construct figure 4 which reveals the major product groups in which the two metals are used.

**Figure 5.**
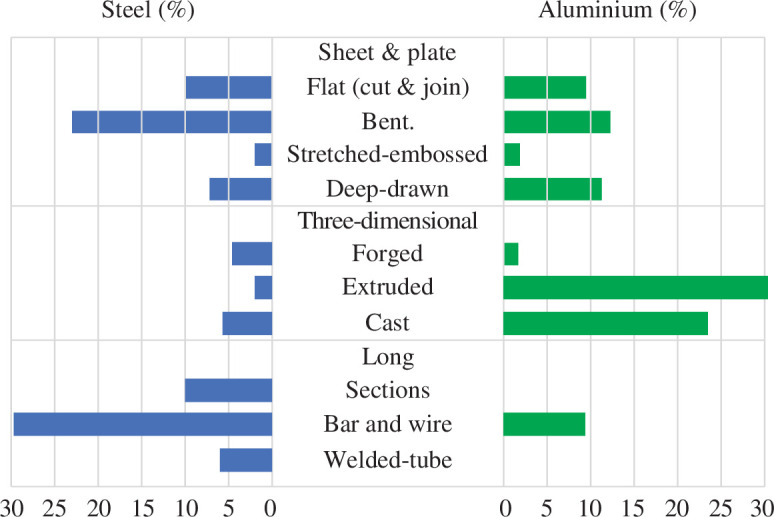
Estimated use of steel and aluminium in components in 2023 [26].

This figure reveals ‘responsibility’ for the use of metal among products but is insufficient to reveal where change could occur: the fact that 5% of steel is used to make cars does not inform an exploration into the question ‘how could we make cars with less steel?’ Accordingly, in work preparatory to this article [26], we made a detailed examination of the component composition of each of the product types in figure 4. We identified 10 fundamentally distinct forms of component, linked to the last high-throughput metal-forming process by which they were produced. For each product type, we derived an estimate of how much metal was used in the form of each component type and scaled it by consumption in 2023. The result of this detailed work is reproduced in figure 5.

The figure groups metal-use into three large categories of sheet and plate, three-dimensional and long components, reflecting the major processes used to produce the components. Sheet, plate and long components are largely made in quasi-continuous production by rolling, while forging, extrusion and casting are inherently discrete.

After these major forming processes, almost all components are subject to some machining activities—including cutting to length, machining precise geometric details and cutting out joints—which remove metal as swarf. However, instead of machining, the classification points towards familiar types of high-throughput metal-forming process, each of which targets specific geometric forms. It provides a starting point for exploring the extent to which metal is used well.

The figure gives clear priority to particular forms of metal use. Bent shapes, such as lightweight sections made out of sheet and used largely in construction, are a large use. Formed sheets that become car body parts or at a larger scale the plates that are welded into the sides of ships are also important. Hot rolled steel sections matter, as does the reinforcing bar used in making reinforced concrete. In contrast, the figure does not prioritize steel or aluminium used in electronic applications, used in powder form or for jewellery, for example, as these all have low volume.

The figure prioritizes deep-drawing for both metals, yet the global analysis of steel [16] demonstrates that deep-drawing has a poor yield: around half of the sheet metal used to make deep-drawn parts is cut off in manufacturing. Primarily this is because during deep-drawing, the metal must be gripped in a blank-holder during the press-stroke, to control feed and navigate a path between the two failure modes of tearing and buckling. After deep-drawing, the part is trimmed, and all material associated with this blank-holder is cut off. This causes most of the scrap, which is recycled, so has been celebrated by manufacturers as part of a ‘circular economy’. However, it would always cause lower emissions to avoid creating the scrap in the first place [27].

**Figure 6.**
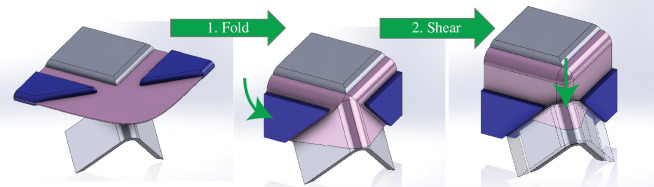
The DeepForm process of folding-shearing.

Inspired by our recognition of this specific material inefficiency, we re-examined the process of deep-drawing and in 2015 invented the process of folding-shearing [28–30] illustrated in figure 6.

First the sheet metal is folded, like origami, to approximate the final part geometry while gathering excess metal in conical shapes at the perimeter. Unlike the flat sheet, these cones (or ‘beaks’) have stiffness, exactly like partially formed components in metal-spinning. Therefore, in a second step, it is possible to push against the stiff ‘beak’ to shear the metal into final shape, without the requirement of a blank holder to grip the edge of the workpiece.

Folding-shearing, motivated by saving metal, is now being commercialized by the company DeepForm Ltd in the UK, which is attracting substantial interest from customers. The invention of folding-shearing arose from recognition of the high volume of scrap created by deep-drawing. The goal of this article is to characterize and explore the scrap associated with all the other component forms in figure 5, to inspire further innovation to reduce future demand for metal.

## 3. Effectiveness: how well do we use metal?

On beginning the analysis for this article, we anticipated making a detailed estimate of the manufacturing scrap for each component type identified in our previous work. However, while doing so, we recognized two further inefficiencies in contemporary use of metal. In many applications, particularly in construction, products are over-specified and in service will never experience loads close to those defined by material limits. For convenience, we call this *specification scrap*. In addition, we noticed that even if some part of a component is used to the limit of its properties, in many cases much of the metal in the component will experience lower loads. For example, for any component loaded in bending, the highest stress occurs only at the greatest distance from the neutral axis of bending. All other material in the bent component is under-exploited, and we call this *property scrap*.

Each of these forms of scrap is now explored in turn.

### (a) Manufacturing scrap

In previous analyses of the global transformation of steel [21] and aluminium [22] into final goods, we have estimated manufacturing yield ratios (the fraction of input material converted into intermediate or final goods in each process in a supply chain) for most of the major processes of metal production. The yield ratios of upstream processes were deduced from balancing mass flow data, reported in previous research [31]. For the downstream processes, we conducted informal interviews with manufacturing engineers in practice to gain their estimates of yield in practice and then verified these by further literature search.

A recent article examined manufacturing yields from use of the deep-drawing process in the automotive sector [32], finding that on average, the industry uses just 56% of all sheet metal it purchases to make cars. The remaining 44% is cut off as manufacturing scrap. Using an estimate of 78% for blanking losses [21], this implies a yield of 72% from deep-drawing and we assume this is representative of other deep-drawing applications. We have failed to identify studies of the yield ratios of stretch-embossing (crash-forming) so assume that manufacturing yield losses are half those of deep-drawing, as the process is used for simpler geometries. Manufacturing losses in forging arise from flash and oxide scale, in addition to defects due to process errors. Two recent case studies of forging a talar body prosthesis [33] and a differential spider [34] report normal yield ratios of 66 and 75%, respectively. These two references also point to two textbooks reporting normal yield ratios in the range 50–90% [35] and 85–90% [36] depending on part geometry. From this survey, we have chosen an estimated yield ratio for forging of 75%. The yield ratio for extrusion/wire drawing is taken from [22], based on an earlier detailed mass-flow analysis of aluminium flows in Europe in 2008. No global estimate of the yield of machining processes is available, and we have identified published case studies of an aluminium wing skin panel [37] (29% yield), a test part based on cylinders [38] (18%) and a thin-walled flared cylinder [39] (11%), but these are likely to be extreme cases, used to motivate the development of additive and other new processes. In contrast, a paper in 2006 estimated that the global mass of dry steel swarf produced annually was 2.3–5.8 Mt, based on data reporting annual global use of 339 million gallons of metal cutting fluids [40]. If we assume that most swarf comes from machining forged metal, we can estimate the yield of machining by estimating the mass of forgings in 2006. The total global mass of steel forgings in 2023 is estimated to be 22 Mt [26]. Global steel production in 2006 was approximately 66% of that today (data from WorldSteel), suggesting about 15 Mt of forgings at the time, so comparing this with the estimate of swarf, suggests a manufacturing yield for machining of approximately 60–85%. With higher values for the case study parts and lower from the overall estimate, but recognizing the high uncertainty of both, we assume that machining has a yield of 60% ± 25%.

Using these figures, table 1 presents our estimate of the yield ratios of the major production processes required to create final steel and aluminium goods. The yield ratios for the two metals are different up to the completion of the intermediate goods supplied to downstream manufacturing and construction, but thereafter the two materials are processed with similar efficiency.

**Table 1.** Estimated yield ratios for the major process steps in making components from steel or aluminium.

process steps		steel	aluminium
1: liquid metal	ore/scrap to refined liquid metal	87%	86%
2: casting	a.slab/bloom/billet casting	96%	91%
	*b.foundry casting of products*	66%	67%
3: hot rolling	a.section rolling	90%	
	b.rod and bar rolling	94%	
	c.plate rolling	90%	85%
	d.hot strip mill	95%	85%
4: cold rolling/welding	a.seamless tube	92%	
	b.welded pipe	94%	
	c.cold rolling including coating	94%	82%
cutting/blanking	a.cutting sections or rebar to length	95%	
	b.cutting bar or rod to shape	88%	
	c.cutting flat plate or blanking sheet	78%	
	d.cutting pipe to length	95%	
shaping	a.stretch-embossing (crash forming)	89%	
	b.deep-drawing and trimming [32]	72%	
	c.forging	75%	
	d.extrusion/wire drawing	76%	
machining	a.cutting parts from bar and forgings	60%	

Unsurprisingly, the table demonstrates that product casting and downstream machining operations have the worst yield ratio.

The components of figure 5 are made in process chains, as liquid metal is cast, generally rolled, then shaped and cut to final design. In order to estimate overall yield ratios for the components, table 2 reports typical process sequences for transforming liquid metal into final parts. By multiplying together the estimated process yield ratios from table 1 in the sequences of table 2, we deduce an overall estimated yield ratio for each component.

**Table 2.** Manufacturing yield ratios for the component catalogue.

component:	steel	aluminium	process sequence
flat—cut and joined	58%	51%	1–2 a−3c−5c
bent sheet	55%	42%	1–2 a−3c−4c−5c
stretched-embossed	49%	38%	1–2 a−3c−4c−5c−6a
deep-drawn	39%	30%	1–2 a−3c−4c−5c−6b
forged	31%	31%	1–2 a−3b−5b−6c−7
extruded	69%	52%	1–2 a−3b−4a−5d, 1–2 a−5b−6d
cast	57%	58%	1−2b
sections	71%	N/A	1–2 a−3a−5a
bar and wire	74%	52%	1–2 a−3b−5a, 1–2 a−5b−6d
welded tube	70%	N/A	1–2 a−3d−4b−5d

Table 2 demonstrates that yield ratios are highest for long products with constant cross-sections, which are largely rolled (or extruded) to shape, and then cut to length. In most sheet metal applications, around half the liquid metal is removed before the final part is completed. The estimated yield ratios are lowest for forged parts, which are generally machined after forging.

### (b) Specification scrap

All designers verge towards caution, and the degree to which they add extra material has for a long time been characterized by ‘Factors of Safety’. Early studies comparing component limit states to expected service loads reported Factors of Safety typically in the range 4–40 [41].

However, over time, more reflective research has revealed that large Factors of Safety are at least partially explained by designers’ uncertainty about their task [42–44]. They may be compelled to add material to their designs to compensate for uncertainty about material properties or the reliability of property tests, about the loads products will experience in both planned and unplanned use, about the accuracy of the models used to predict limit states, about stress localization, the consequences of manufacturing imperfections or to anticipate the possibility of changes in the client brief. Designers must also aim to anticipate future product evolutions [45], for example with ‘platform designs’, including deliberate redundancy in an early design, to enable more rapid design of future variations [46].

Factors of safety that address all of these forms of uncertainty are essential for product safety and for many products have become codified in product standards. However, on top of this defence against uncertainty, designers often over-specify their products further. In judging how much additional material to use, they weigh the costs of further design time and manufacturing complexity against the cost of material addition, and for steel and aluminium, which are produced in huge volumes with tremendous economies of scale, it will often be economically rational, under present regulatory and cost structures, to trade-off increased material use against reduced design or manufacturing time.

However, that economic rationale does not align with targets to reduce demand for the two metals in the pursuit of a zero-emissions economy.

To date, there are few studies which explicitly examine over-design. Two examples, which examine real-world case studies of steel-framed commercial buildings in the UK, reveal that over-specification—that is the use of material over and above what is required by the already conservative Eurocodes that govern European building standards—averages between 50% [47] and 100% [48]. This applies mainly to steel sections, so gives a sensible basis for estimating global over-specification of components of that form.

In the absence of similar studies for the other components in figure 5, table 3 presents an estimate and a likely range for the over-specification of steel and aluminium across the 10 component types, justified by specific case studies. There is significant uncertainty behind these estimates, and in time, we can anticipate that future researchers will add resolution and precision to the numbers.

**Table 3.** Over-specification for the component catalogue.

component:	estimated over-specification	range	case studies in literature
flat—cut and joined	1.5	1.1–2.5	container ship FoS = 1.3 for ultimate stress [49]
bent sheet	1.5	1.1–2.5	FoS = 1.4 for agricultural trailer [50]
stretched-embossed	1.5	1.1–2.5	*assumed the same as flat or bent sheet*
deep-drawn	3.0	1.5–5.0	chemical engineering evaporator [51]
forged	2.0	1.5–3.0	analyses of drive shafts [52,53]
extruded	1.5	1.2–2.0	curtain wall structure under wind load [54]
cast	3.0	1.5–5.0	*assumed 50% higher than forgings*
sections	1.75	1.5–2.5	steel-framed buildings [11,32], cantilevered tube [53]
bar & wire	1.15	1.1–1.5	currently recommend for re-bar in the UK [55]
welded tube	2.5	2.0–3.0	offshore structures FoS~2 [56], line pipe FoS = 3 [57] crash-structures [58,59]

Although table 3 is subject to high uncertainty, and offers no differentiation between the two metals, and two of the numbers have been estimated by similarity with other components in the absence of any literature, the reality of over-specification is widely acknowledged. For the purposes of this article, these first estimates will therefore be used as a pointer to the scale of savings in material use which might be achieved once supply falls substantially short of demand.

### (c) Property scrap

A wire loaded in uniaxial tension experiences the same stress everywhere, so in theory, as the load on the wire is increased, the whole volume of material will reach the property limit of the yield stress at the same time. This is an example of a component loaded with no ‘property scrap’ for strength. It is likely that other properties of the wire are not fully exploited, and it is probably impossible to avoid any scrap in every property of the material. However, material selection (as exemplified by Ashby [60], for example) typically focuses on one or two dominant properties, and our interest in ‘property scrap’ is to consider to what extent these dominant properties are exploited.

**Figure 7.**
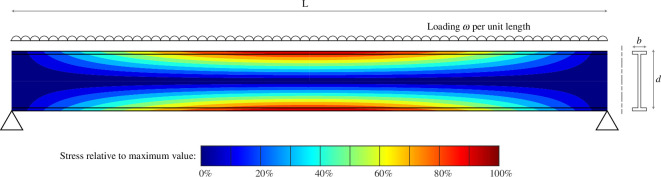
Relative stress in a simply supported I-beam.

In contrast, even when designed without any overspecification, the material properties in most components are used less efficiently, as the stress in the material is not uniform but distributed. This is illustrated in figure 7 for a simply supported I-beam subject to uniform loading.

The bending moment distribution varies along the length of the beam, and the stress varies with distance from the neutral axis, so using simple beam theory, the longitudinal stress in the beam is:


(3.1) $$\sigma \left(x,y\right)=-\frac{wy}{2I}\left(L^{2}-x^{2}\right).$$

If the limit to beam capacity is defined by the loading at which this stress first reaches yield (in the centre of the beam, at the furthest distance from the neutral axis), the stress everywhere else is lower than this limit, as illustrated by the coloured shading in figure 7. Integrating one minus the ratio of this stress to the yield stress, over the volume of the beam in figure 7 and dividing by volume, gives the average property scrap fraction,Ψ , as:


(3.2) $$\psi =\frac{2\left(d+b\right)}{3\left(d+2b\right)}.$$

For a typical I-beam with *d* = 4*b*, the ‘property scrap’ defined as the fraction of un-used material capacity (i.e. stress below maximum capacity), is therefore 56%. Similar analysis applied to solid circular shafts under torsion gives an average property scrap of 33%, motivating the selection of hollow shafts where possible [61].

This analysis demonstrates that property scrap is distinct from specification scrap. The simple beam illustrated in figure 7 would be over-specified if it had a second moment of area that would never be required by the loads experienced in service. However, even if it is specified perfectly, the figure demonstrates that it has substantial property scrap because the loading arrangement cannot exploit the full benefit of the metal’s properties across the full volume of the component.

**Figure 8.**
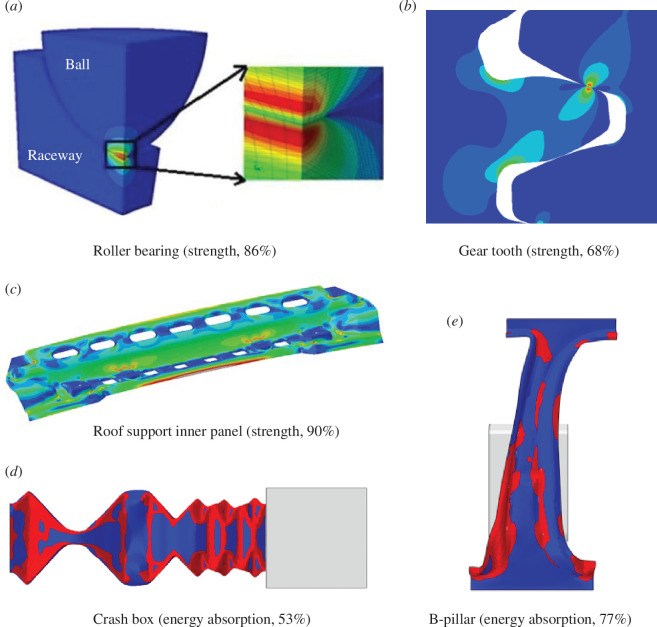
Finite-element analysis of the limit states of five representative components, showing the limiting property in brackets and resulting property scrap fraction.

To extend the approach of the simple analysis in equation (3.1), (3.2) across the component catalogue of figure 5, we conducted finite-element analyses of the limit states of five representative components, as illustrated in figure 8. The analysis in the figures was conducted with the commercial software package Abaqus. For the crash-box and B-pillar, a dynamic analysis was performed with Abaqus Explicit, using explicit time integration without time or mass-scaling and with quadrilateral shell elements having 7 integration points through the thickness and sized to have at least 5 elements along each fold or radius. The roof support inner panel was analysed with quasi-static, implicit time integration with quadrilateral shell elements and a similar mesh. The gear and bearing were analysed with a quasi-static, implicit time integration scheme with quadrilateral full integration continuum plane strain (gear) or brick (bearing) elements and a variable mesh was designed to have more elements in the areas of high stress concentration at the point of contact. As the figure is intended to convey distributions, specific geometric and loading parameters have not been given, and scales are not shown. For the strength cases (a–c), the colour scale is of equivalent stress, with red for the highest value. For the two energy-absorbing cases, the colour scale is of equivalent plastic strain and has been simplified to show ‘high’ (red) and ‘low’ (blue) levels.

The results of figure 8 are clearly challenging to designers, who for each component, have sought mechanical efficiency. Nevertheless, for each component, the figure demonstrates that more than half the metal is not used to the limits of its properties. The high contact stresses in the bearing (a) and gear (b) demand the greatest performance of the metal, yet these stresses are highly localized, so most of the metal elsewhere in the component is loaded well below its potential. The roof support panel (c), designed primarily for strength when a car rolls over, is mainly loaded in bending, so like the beam in figure 7 is mainly not used to capacity. For the two safety components (d and e), the design aims to absorb energy, but once plastic deformation has begun, it becomes localized in the folds and hinges of plastic collapse, so the rest of the metal absorbs no energy. A study on corrugated structures buckling under axial compression provides some support for this estimate by revealing the range of performance possible for different designs [62].

The analyses in figures 7 and 8 are no more than indicative but provide a basis for compiling a first set of estimates of property scrap, across the catalogue of components in figure 5. This is presented in table 4, with a nominal value of property scrap for each component and a possible range. In future, the analysis could be extended to consider all material properties under all load-cases.

**Table 4.** Estimated property scrap across the component catalogue.

component:	estimated property scrap (%)	range	notes
flat—cut and joined	60	40–80	assumed loaded mainly in bending
bent sheet	60	40–80	mainly sections/corrugations in bending
stretched-embossed	90	60–95	based on figure 8*c*
deep-drawn	60	50–80	based on figure 8*d*,*e*
forged	50	30–80	based on figure 8*a,b* and torsion shaft
extruded	60	40–80	mainly loaded in bending
cast	50	30–80	same as forged
sections	60	40–80	based on figure 7 and equation (3.2)
bar & wire	20	10–50	largely loaded uniaxially
welded tube	60	40–80	mainly loaded in bending

The message of table 4 is that other than for uniaxially loaded wire or reinforcing bars, it is extremely difficult to design metal components to make full use of their properties. This would have been familiar to our forebears who, for example, used metal only to create tough tips for their wooden farming tools, to make the best use of their limited supply of metal. Potentially this evidence about property inefficiency suggests that we might aim to tailor properties to reflect product design requirements, and a rich seam of science and technology innovations aims to do this [63]. However, while this approach might reduce the property-scrap reported in the table, it would do little to reduce the emissions of production, which are related primarily to liquid metal production, and not the downstream actions of deformation and temperature control that shape the metal and govern its microstructure.

## 4. Results

**Figure 9.**
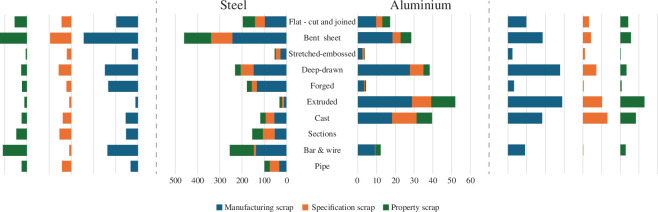
Three forms of ‘scrap’ in 2023 use of steel and aluminium, by component type.

Tables 2–4 now allow creation of the main result of this article, when applied to the global use of the two metals in components as reported in figure 5, with data scaled to match total global use in 2023 [19]. This leads to the values in table 5 summarized in the exploded bar charts of figure 9. Starting from final use, the yield ratios in table 2 are used to calculate the upstream production scrap from liquid metal to final goods. The calculation of specification scrap and property scrap depends on the sequence in which the ratios of tables 3 and 4 have been applied, so to avoid prejudicing the results in either direction, have been allocated in proportion to their logarithms.

**Table 5.** Estimated inefficiency of steel and aluminium use by component type (Column headings: A – final use in 2023; B – manufacturing scrap; C – specification scrap; D – property scrap; E – essential use).

	steel					aluminium				
*all values in Mt/yr*	*A*	B	C	D	*E*	*A*	B	C	D	*E*
flat—cut and joined	134	97	43	55	36	7	7	2.2	2.8	1.8
bent sheet	297	243	96	121	79	9	12	2.9	3.7	2.4
stretched-embossed	27	28	20	5	2	1	2	0.7	0.2	0.1
deep-drawn	95	149	56	26	13	8	19	4.7	2.2	1.1
forged	60	134	23	23	15	1	2	0.4	0.4	0.3
extruded	26	12	8	11	7	21	19	6.8	8.6	5.6
cast	74	56	38	24	12	17	12	8.7	5.5	2.8
sections	130	53	52	48	30					
bar & wire	389	137	9	109	271	7	6	0.2	1.9	4.7
pipe	78	33	42	23	12					
**totals**	**1310**	**941**	**389**	**445**	**476**	**71**	**79**	**27**	**25**	**18**

The sum of column A is the reported final use, so when added to the sum of column B is the sum total of all liquid metal production. This total is greater than that reported by the two industries, because they do not report internal recycling or losses at the liquid stage. The value for ‘Essential use’ in column E is the residual once property and specification scrap have been removed from final use.

Overall, our results suggest that 80% of all steel and 90% of all aluminium produced in 2023 were not essential. This bold and striking statistic should be a wake-up call in climate policy, and in the industrial, innovation and research communities, to re-examine their assumptions about future metal demand. The figures are, of course, subject to significant uncertainty, but present a broad truth: the bulk metals are made in such volume and at such low cost, that we have become careless about their use.

For both metals, manufacturing scrap is the greatest cause of material inefficiency, equivalent to two-thirds of final use for steel, and exceeding final use of aluminium. Yet both other forms of scrap are important. Final use could be more than halved if both these inefficiencies were eliminated, and the net benefit would propagate upstream with proportional reductions in manufacturing losses.

For aluminium, a clear priority is to target the use of sheet metal, particularly in deep-drawn applications. Most such use is in production of cars and light vans, with a smaller contribution from drinks cans, where blanking scrap is significant. Extruded aluminium used in construction and the production of industrial equipment is equally a target, and potentially the highest priority for reducing property scrap.

For steel, by far the dominant cause of inefficiency is in bent sheet, used for light-weight sections, corrugated sheets and other load-bearing components, primarily in construction. This is a striking revelation, as such use receives so little attention. The fact that it has such importance suggests that this could be a ripe area for innovation. Despite the relative efficiency of its specification and use of properties, bar and wire applications are also a significant source of loss, as these forms of component are the largest use of steel.

## 5. Discussion

The results of this article are important and striking and inevitably open to challenge. In the absence of detailed data, we have found the best available sources to make estimates of inefficient metal use. These are subject to uncertainty, which we have estimated to be approximately ±50% for each form of scrap. The overall scrap rates of 80% for steel and 90% for aluminium were calculated from table 5 with the formula (B + C + D) / (A + B) applied to the row sums, using the given column headings. Allowing the totals for three types of scrap, B, C and D, to vary in the range ±50% gives ranges for the total scrap rates as 64–98% for steel and 80–95% for aluminium. Even at the lowest ends of this range, our numbers reinforce the fact that we could live well with much less metal, by using it more wisely.

In compiling this first set of estimates, we have considered only single-load cases. In reality, most components must meet not only multiple load cases but also multiple design criteria (e.g. for both stiffness and strength) and we cannot anticipate how this will influence the scale of our results.

The three forms of scrap examined in the article are multiplicative, and any increase in property or specification scrap will lead to an increase in manufacturing scrap. While the results demonstrate that manufacturing scrap has the largest total, this multiplication motivates attention on all three forms equally. Similarly, propagating the results further upstream, any reduction in liquid metal demand will disproportionately reduce high-emitting primary production, as producing liquid metal by recycling scrap will always require less energy.

Around 85% of global demand for these two metals is for commercial construction and infrastructure, industrial equipment, non-private vehicles and other goods. As a result, final consumer choice and preferences will have little impact as a driver of demand reduction. These two metals are primarily purchased in business-to-business transactions, and therefore, the ambitions of downstream producers to drive down the embodied emissions of their products can be a powerful force for change.

In response to the inefficiencies revealed in this article, we provide tentative design guidance targeted on the three forms of scrap.

Design principles to avoid property scrap:

— Avoid bending where possible, by realigning loads with the structure and using truss-like load-bearing structures to align forces with structural elements.
— Aim at designs with uniform stress states, through managing loads and different structural forms, and where this is not possible, seek to re-shape the structure to make better use of less metal in areas that currently experience lower load.
— Seek opportunities in material selection to use lower embodied emissions materials than metal when the full properties of metal are not required.

Design principles to avoid specification scrap:

— Where existing design codes and standards specify factors of safety to compensate for uncertainty, or dynamic loading, aim at forms of redesign to reduce variability—for example through controlling static load variation by changing user options, or using dynamic compensation.
— Make increased use of sensors in structures, both to generate an insight into future designs and to ensure safety in more efficient designs, by giving warning about approaching limits.
— In structures governed by both stiffness and strength limits, aim to design for strength and then apply other dynamic compensation or actuation for stiffness.

Principles for finding valuable process innovations in manufacturing:

— Focus on adjusting high volume processes, for example with new tooling or additional actuators, in order to maintain throughput. Decades of research on novel forming processes [64] have traded flexibility with throughput, but to reduce overall demand, throughput is the key. Additive and powder processes use little metal but are energy intensive and slow [65], so can play no significant role in reducing demand. Instead, it is likely that the innovations which can change metal demand at scale will be adjustments to the tooling or controls of the familiar high volume processes prioritized by the catalogue of figure 5. The DeepForm innovation in figure 6 and the invention of tailor-rolled blanks in Aachen exemplify such innovations in tooling and control. The material benefits of saving metal in component production should then be evaluated against the additional material required to adapt or replace tooling.
— Shine a new light on the use of bent sheets, particularly in the construction industry, to reduce all three forms of scrap, by placing material only where it is essential.
— Re-examine the opportunity for multi-material solutions, particularly for shaped sheets, which generally have low stiffness, and for welded pipe, where the loads during installation are often greater than those in service.

## 6. Conclusion

It is virtually impossible that the volume of today’s highly emitting supply of steel and aluminium can be made with zero emissions by 2050. As a result, meeting targets for zero emissions by that date depends on using substantially less metal. For the first time, this article has estimated the opportunity to use less metal, at a component level, in order to direct the attention of designers and manufacturing technologists to the applications where they can have most impact. Despite high uncertainty in all of the estimates used in the article, the results overall suggest that around 80% of steel and 90% of aluminium liquid metal production may not have been required to deliver designed functions. This motivates urgent and in-depth examination of the specific opportunities to use less metal, particularly for sheet metal components and extruded aluminium.

## Data Availability

All data used in the article are presented in the tables within the manuscript.

## References

[1] Climate Change Committee. 2023 Climate change committee: progress in reducing emissions. Report to Parliament. See https://www.theccc.org.uk/ (accessed 6 May 2024).

[2] Smil V. 2014 The long slow rise of solar and wind. Sci. Am. **310**, 52–57. (10.1038/scientificamerican0114-52) 24616971

[3] Gross R, Hanna R, Gambhir A, Heptonstall P, Speirs J. 2018 How long does innovation and commercialisation in the energy sectors take? Historical case studies of the timescale from invention to widespread commercialisation in energy supply and end use technology. Energy Policy **123**, 682–699. (10.1016/j.enpol.2018.08.061)

[4] Nelson S, Allwood JM. 2021 The technological and social timelines of climate mitigation: lessons from 12 past transitions. Energy Policy **152**, 112155. (10.1016/j.enpol.2021.112155)

[5] Allwood JM, Cullen JM, Carruth MA, Cooper DR, McBrien M, Milford RL, Patel AC. 2012 Sustainable materials: with both eyes open. vol. 2012. Cambridge, UK: UIT Cambridge Limited.

[6] Allwood JM, Cullen JM, Milford RL. 2010 Options for achieving a 50% cut in industrial carbon emissions by 2050. Environ. Sci. Technol. **44**, 1888–1894. (10.1021/es902909k) 20121181

[7] USGS. 2024 Minerals Yearbook – metals and minerals. See https://www.usgs.gov/centers/national-minerals-information-center/minerals-yearbook-metals-and-minerals (accessed 6 May 2024).

[8] International Energy Agency. 2024 Average GHG emissions intensity for production of selected commodities. See https://www.iea.org/data-and-statistics (accessed 6 May 2024).

[9] ING. 2021 Metals & mining decarbonisation and sector disclosure. See https://think.ing.com/articles (accessed 6 May 2024).

[10] Yokoi R, Watari T, Motoshita M. 2022 Future greenhouse gas emissions from metal production: gaps and opportunities towards climate goals. Energy Environ. Sci. **15**, 146–157. (10.1039/D1EE02165F)

[11] Kim J, Sovacool BK, Bazilian M, Griffiths S, Lee J, Yang M, Lee J. 2022 Decarbonizing the iron and steel industry: a systematic review of sociotechnical systems, technological innovations, and policy options. Energy Res. Soc. Sci. **89**, 102565. (10.1016/j.erss.2022.102565)

[12] Pedneault J, Majeau-Bettez G, Krey V, Margni M. 2021 What future for primary aluminium production in a decarbonizing economy? Glob. Environ. Change **69**, 102316. (10.1016/j.gloenvcha.2021.102316)

[13] Gast L, Allwood JM. 2023 What bulk material production is possible on a transition to net zero emissions by 2050 with limited zero emissions resources? J. Clean. Prod. **423**, 138346. (10.1016/j.jclepro.2023.138346)

[14] Watari T, McLellan B. 2024 Decarbonizing the global steel industry in a resource-constrained future—a systems perspective. Phil. Trans. R. Soc. A (10.1098/rsta.2023.0233) PMC11542903 39489167

[15] Hafez H, Drewniok MP, Velenturf APM, Purnell P. 2024 A resource-bound critical analysis of the decarbonisation roadmaps for the UK foundation industries by 2050. Environments **11**, 153. (10.3390/environments11070153)

[16] World Steel Association. 2022 Sustainability indicators 2022 report. Sustainability performance of the steel industry 2003-2021. See https://worldsteel.org/ (accessed 6 May 2024).

[17] Lamb WF *et al*. 2021 A review of trends and drivers of greenhouse gas emissions by sector from 1990 to 2018. Environ. Res. Lett. **16**, 073005. (10.1088/1748-9326/abee4e)

[18] Lee WS, Lee JC, Oh HT, Baek SW, Oh M, Lee CH. 2017 Performance, economic and exergy analyses of carbon capture processes for a 300 MW class integrated gasification combined cycle power plant. Energy (Oxf). **134**, 731–742. (10.1016/j.energy.2017.06.059)

[19] BP. 2022 BP Statistical Review of World Energy 2022. See https://www.bp.com/ (accessed 6 May 2024).

[20] Liu G, Bangs CE, Müller DB. 2013 Stock dynamics and emission pathways of the global aluminium cycle. Nat. Clim. Chang. **3**, 338–342. (10.1038/nclimate1698)

[21] Cullen JM, Allwood JM, Bambach MD. 2012 Mapping the global flow of steel: from steelmaking to end-use goods. Environ. Sci. Technol. **46**, 13048–13055. (10.1021/es302433p) 23167601

[22] Cullen JM, Allwood JM. 2013 Mapping the global flow of aluminum: from liquid aluminum to end-use goods. Environ. Sci. Technol. **47**, 3057–3064. (10.1021/es304256s) 23438734

[23] Yang H, Ma L, Li Z. 2023 Tracing China’s steel use from steel flows in the production system to steel footprints in the consumption system. Renew. Sustain. Energy Rev. **172**, 113040. (10.1016/j.rser.2022.113040)

[24] Zhu Y, Syndergaard K, Cooper DR. 2019 Mapping the annual flow of steel in the United States. Environ. Sci. Technol. **53**, 11260–11268. (10.1021/acs.est.9b01016) 31468962

[25] Reck BK, Zhu Y, Althaf S, Cooper DR. 2024 Assessing the status quo of US steel circularity and decarbonization options. Tech. Innov. for the Circ. Econ. Recycl. Remanuf. Design Syst. Anal. and Logist. 211–221. (10.1002/9781394214297)

[26] Music O, Allwood JM. 2024 Connecting environmental systems analysis to manufacturing technology: a catalogue of the world’s steel and aluminium components. Resour. Conserv. Recycl.

[27] Horton P, Allwood J, Cassell P, Edwards C, Tautscher A. 2018 Material demand reduction and closed-loop recycling automotive aluminium. MRS Adv. **3**, 1393–1398. (10.1557/adv.2018.280)

[28] Allwood JM, Cleaver CJ, Loukaides EG, Music O, Nagy-Sochacki A. 2019 Folding-shearing: shrinking and stretching sheet metal with no thickness change. CIRP Ann. **68**, 285–288. (10.1016/j.cirp.2019.04.045)

[29] Cleaver CJ, Arora R, Loukaides EG, Allwood JM. 2022 Producing isolated shrink corners by folding-shearing. CIRP Ann. **71**, 217–220. (10.1016/j.cirp.2022.03.036)

[30] Arora R, Music O, Cleaver CJ, Allwood JM. 2023 An exploration of the process operating window for folding-shearing in a press-tool. In International conference on the technology of plasticity, pp. 70–77. Cham, Switzerland: Springer Nature. (10.1007/978-3-031-40920-2_8)

[31] Hatayama H, Daigo I, Matsuno Y, Adachi Y. 2010 Outlook of the world steel cycle based on the stock and flow dynamics. Environ. Sci. Technol. **44**, 6457–6463. (10.1021/es100044n) 20704247

[32] Horton PM, Allwood JM. 2017 Yield improvement opportunities for manufacturing automotive sheet metal components. J. Mater. Process. Technol. **249**, 78–88. (10.1016/j.jmatprotec.2017.05.037)

[33] Soranansri P, Rojhirunsakool T, Nithipratheep N, Ngaouwnthong C, Boonpradit K, Treevisootand C, Srithong W, Chuchuay P, Sirivedin K. 2021 Hot forging process design and initial billet size optimization for manufacturing of the talar body prosthesis by finite element modeling. j.asep (10.14416/j.asep.2021.01.002)

[34] Prithvi Raj M, Kumar M, Pramanick AK. 2020 Yield improvement in hot forging of differential spider. Mater. Today **26**, 3107–3115. (10.1016/j.matpr.2020.02.642)

[35] Tschaetsch H. 2006 Impression-die forging (closed-die forging). Met. Form. Pract. 123–140. (10.1007/3-540-33217-0_13)

[36] Handbook ASM. 1996 Volume 14: forming and forging. Ohio: ASM International, Novelty.

[37] Milford RL, Allwood JM, Cullen JM. 2011 Assessing the potential of yield improvements, through process scrap reduction, for energy and CO_2_ abatement in the steel and aluminium sectors. Resour. Conserv. Recycl. **55**, 1185–1195. (10.1016/j.resconrec.2011.05.021)

[38] Wippermann A, Gutowski TG, Denkena B, Dittrich MA, Wessarges Y. 2020 Electrical energy and material efficiency analysis of machining, additive and hybrid manufacturing. J. Clean. Prod. **251**, 119731. (10.1016/j.jclepro.2019.119731)

[39] Liu Z, Zhao Y, Wang Q, Xing H, Sun J. 2024 Modeling and assessment of carbon emissions in additive-subtractive integrated hybrid manufacturing based on energy and material analysis. Int. J. of Precis. Eng. and Manuf.-Green Tech. **11**, 799–813. (10.1007/s40684-023-00588-3)

[40] Chang JI, Lin JJ, Huang JS, Chang YM. 2006 Recycling oil and steel from grinding swarf. Resour. Conserv. Recycl. **49**, 191–201. (10.1016/j.resconrec.2006.03.014)

[41] Huff R. Factors of safety. Transactions (Society of Automobile Engineers) 70–87. https://www.jstor.org/stable/44579826

[42] Khurmi RS, Gupta JK. 2005 A textbook of machine design. India: Eurasia Publishing House.

[43] Aughenbaugh JM, Paredis CJJ. 2006 The value of using imprecise probabilities in engineering design. J. Mech. Des. **128**, 969–979. (10.1115/1.2204976)

[44] Eckert C, Isaksson O, Lebjioui S, Earl CF, Edlund S. 2020 Design margins in industrial practice. Des. Sci. **6**, e30. (10.1017/dsj.2020.19)

[45] Allen JD, Stevenson PD, Mattson CA, Hatch NW. 2019 Over-design versus redesign as a response to future requirements. J. Mech. Des. **141**. (10.1115/1.4042335)

[46] Brahma A, Ferguson S, Eckert C, Isaksson O. 2023 Margins in design – review of related concepts and methods. J. Eng. Des. 1–34. (10.1080/09544828.2023.2225842)

[47] Dunant CF, Drewniok MP, Eleftheriadis S, Cullen JM, Allwood JM. 2018 Regularity and optimisation practice in steel structural frames in real design cases. Resour. Conserv. Recycl. **134**, 294–302. (10.1016/j.resconrec.2018.01.009)

[48] Moynihan MC, Allwood JM. 2014 Utilization of structural steel in buildings. Proc. R. Soc. A **470**, 20140170. (10.1098/rspa.2014.0170) 25104911 PMC4075790

[49] Alfred Mohammed E, Benson SD, Hirdaris SE, Dow RS. 2016 Design safety margin of a 10,000 TEU container ship through ultimate hull girder load combination analysis. Mar. Struct. **46**, 78–101. (10.1016/j.marstruc.2015.12.003)

[50] Szulc M, Malujda I, Talaśka K. 2016 Method of determination of safety factor on example of selected structure. Procedia Eng. **136**, 50–55. (10.1016/j.proeng.2016.01.173)

[51] Sigova EM, Doronin SV. 2007 Computed estimate of safety factors for shell elements of production equipment. Chem. Petrol. Eng. **43**, 647–652. (10.1007/s10556-007-0115-8)

[52] Budynas RG, Nisbett JK. Shigley’s mechanical engineering design, p. 409, vol. 9. New York, NY: McGraw-Hill.

[53] Yin J, Du X. 2020 A Practical safety factor method for reliability-based component design. In ASME 2020 international design engineering technical conferences and computers and information in engineering conference, Virtual, Online. (10.1115/DETC2020-22030)

[54] ASTM A. 2014 E330/E330M-Standard Test Method for Structural Performance of Exterior Windows. Doors, Skylights and Curtain Walls by Uniform Static Air Pressure Difference

[55] Beeby A, Jackson P. 2016 Partial safety factor for reinforcement. Struct. **5**, 101–111. (10.1016/j.istruc.2015.09.002)

[56] Stacey A, Sharp JV. 2007 Safety factor requirements for the offshore industry. Eng. Fail. Anal. **14**, 442–458. (10.1016/j.engfailanal.2005.08.003)

[57] Pipes DI, Fittings A. 2009 Their joints for water applications. I.S.O. **2531**.https://www.iso.org/standard/40396.html

[58] Mortazavi Moghaddam A, Kheradpisheh A, Asgari M. 2021 A basic design for automotive crash boxes using an efficient corrugated conical tube. Proc. Inst. Mech. Eng. Part D J. Automob. Eng. **235**, 1835–1848. (10.1177/0954407021990921)

[59] Buschsieweke O, Kettler M, Schroeter M. 2011 Crash box, and method of making a crash box.

[60] Ashby MF. 2016 Materials selection in mechanical design, 5th edn. Oxford, UK: Butterworth Heinemann.

[61] Bawkar A, Ambekar T, Amle P, Bhosale T, Mujumdar A. 2021 Design and fatigue optimization of drive shaft. Int. J. Res. Appl. Sci. Eng. Technol. **9**, 194–203. (10.22214/ijraset.2021.37285)

[62] Ha NS, Lu G. 2020 Thin-walled corrugated structures: a review of crashworthiness designs and energy absorption characteristics. Thin-Walled Struct. **157**, 106995. (10.1016/j.tws.2020.106995)

[63] Tekkaya AE *et al*. 2015 Metal forming beyond shaping: predicting and setting product properties. CIRP Ann. **64**, 629–653. (10.1016/j.cirp.2015.05.001)

[64] Allwood JM, Utsunomiya H. 2006 A survey of flexible forming processes in Japan. Int. J. Mach. Tools Manuf. **46**, 1939–1960. (10.1016/j.ijmachtools.2006.01.034)

[65] Azevedo JMC, CabreraSerrenho A, Allwood JM. 2018 Energy and material efficiency of steel powder metallurgy. Powder Technol. **328**, 329–336. (10.1016/j.powtec.2018.01.009)

